# Effect of Tomato Peel Extract Grown under Drought Stress Condition in a Sarcopenia Model

**DOI:** 10.3390/molecules27082563

**Published:** 2022-04-15

**Authors:** Francesca Felice, Maria Michela Cesare, Luca Fredianelli, Marinella De Leo, Veronica Conti, Alessandra Braca, Rossella Di Stefano

**Affiliations:** 1 Department of Surgical, Medical and Molecular Pathology and Critical Care Medicine, University of Pisa, 56100 Pisa, Italy; rossella.distefano@unipi.it; 2 Department of Life Sciences, University of Siena, Via P.A. Mattioli 4, 53100 Siena, Italy; maria.cesare@student.unisi.it (M.M.C.); conti30@student.unisi.it (V.C.); 3 Institute for Chemical-Physical Processes of the Italian Research Council (CNR-IPCF), Via Moruzzi 1, 56100 Pisa, Italy; luca.fredianelli@ipcf.cnr.it; 4 Department of Pharmacy, University of Pisa, 56126 Pisa, Italy; marinella.deleo@unipi.it (M.D.L.); alessandra.braca@unipi.it (A.B.); 5 Interdepartmental Research Center “Nutraceuticals and Food for Health”, University of Pisa, 56100 Pisa, Italy; 6 CISUP, Centre for Instrumentation Sharing, University of Pisa, 56126 Pisa, Italy

**Keywords:** tomato by-product, drought stress, polyphenols, human skeletal muscle myoblasts, sarcopenia

## Abstract

Tomatoes and their derivates represent an important source of natural biologically active components. The present study aims to investigate the protective effect of tomato peel extracts, grown in normal (RED-Ctr) or in drought stress (RED-Ds) conditions, on an experimental model of sarcopenia. The phenolic profile and total polyphenols content (TPC) of RED-Ctr and RED-Ds were determined by Ultra High-Performance Liquid Chromatography (UHPLC) analyses coupled to electrospray ionization high-resolution mass spectrometry (ESI-HR-MS). Human skeletal muscle myoblasts (HSMM) were differentiated in myotubes, and sarcopenia was induced by dexamethasone (DEXA) treatment. Differentiation and sarcopenia were evaluated by both real-time PCR and immunofluorescent techniques. Data show that myosin heavy chain 2 (MYH2), troponin T (TNNT1), and miogenin (MYOG) were expressed in differentiated myotubes. 5 μg Gallic Acid Equivalent (GAE/mL) of TPC from RED-Ds extract significantly reduced muscle atrophy induced by DEXA. Moreover, Forkhead BoxO1 (FOXO1) expression, involved in cell atrophy, was significantly decreased by RED-Ds extract. The protective effect of tomato peel extracts depended on their qualitative polyphenolic composition, resulting effectively in the in vitro model of sarcopenia.

## 1. Introduction

Over the years, a decrease in muscle mass is a physiological condition that mainly affects athletes who engage in endurance physical activity. The decrease in muscle mass can take on pathological connotations, up to sarcopenia.

Sarcopenia is a multifactorial process, characterized by inflammation, oxidative stress, motor neuron loss, a change in endocrine function and age-related loss of muscle mass and function, due to an increase in muscle protein degradation and reduced protein synthesis [1,2,3,4]. In sarcopenic muscle, a reduction in the number of myofibers and hypotrophic myofibers, as well as infiltration into adipose and, at later stages, fibrotic tissue, has also been observed [5].

Numerous studies have shown that glucocorticoids decrease muscle size, due to accelerated muscle protein breakdown [6,7]. Thus, to mimic in vitro sarcopenia, treatment with DEXA, a synthetic glucocorticoid, is used. In particular, it has been reported as a non-genomic mechanism of action, induced by DEXA in myotube atrophy [8,9,10].

**Figure 1:**
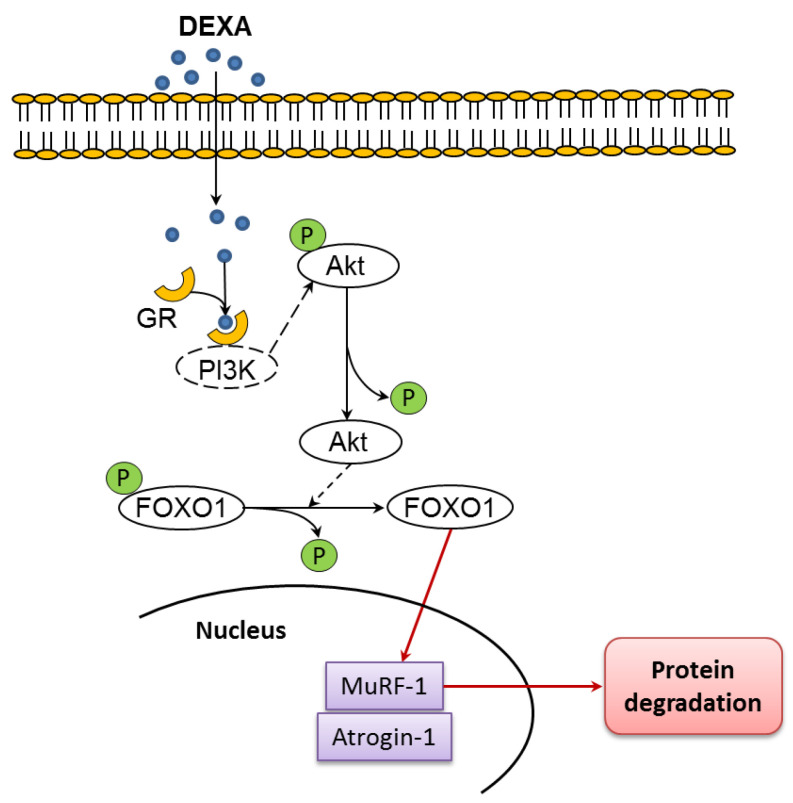
Upregulation of atrogin-1 and MuRF1 by glucocorticoids have been linked to activation of Forkhead BoxO (FOXO1) and FOXO3A resulting from reduced protein kinase B (Akt) activity. Atrogin-1 and MuRF1 are two muscle-specific ubiquitin ligases linked to muscle atrophy when upregulated. Glucocorticoid receptor: GR. Modified by Chen et al. [13].

The degradation of skeletal muscle proteins is controlled by two proteolytic pathways: the ubiquitin–proteasome system and autophagy. Recently, FOXO transcription factors have been identified as the main coordinators of these proteolytic pathways, by virtue of their capacity to induce several autophagy-related genes, as well as the ubiquitin ligases atrogine-1 and muscle ring-finger 1 (MuRF1) [11,12], (Figure 1).

Sarcopenia can be counteracted with a change in eating habits, as a way to overcome anabolic resistance of the muscle. Oral antioxidant supplementation may contribute to reducing the indices of oxidative stress, both in animal and human models, by reinforcing the natural endogenous defenses [14]. The use of antioxidants has been shown to be efficient against sarcopenia, preventing or delaying skeletal muscle atrophy [15,16,17]. Among natural antioxidants, polyphenols constituted the most abundant compounds in the diet and can be divided into different classes, such as phenolic acids, flavonoids, stilbenes, and lignans. These antioxidants are classified according to the number of phenol rings and of the structural elements that bind these rings to one another. 

Tomato (*Solanum lycopersicum* L.) is one of the most consumed vegetables worldwide, both fresh or processed, and it is characterized by a high content of polyphenols. The tomato adapts to different climatic conditions and the abiotic stress occurrence, such as water stress, can represent one of the biggest problems for this crop cultivation, together with the reuse of its by-products, which represent a significant source of natural antioxidants in the human diet [18,19]. Recently, authors demonstrated the antioxidant properties on vascular-related dysfunction of peel extracts from Rosso di Pitigliano tomatoes, an ancient Tuscan tomato variety, obtained by growing plants in normal or in drought stress conditions [20].

The present study aims at evaluating the protective properties of tomato peel polyphenols from Rosso di Pitigliano, cultivated in normal or in drought stress conditions on an in vitro model of sarcopenia. Firstly, human skeletal muscle cells (HSMM), for their capacity to reproducibly differentiate into multinucleated myotubes, were examined. Myoblasts differentiated into myotubes were studied for the expression of various markers of skeletal muscle cells, such as MYH2, TNNT, and MYOG, and by immunofluorescent techniques. Differentiated cells were exposed to DEXA treatment and the protective effect of tomato peel extract was evaluated.

## 2. Results

### 2.1. Characterization and Phenolic Profile of Tomato Peel Extracts

**Figure 2:**
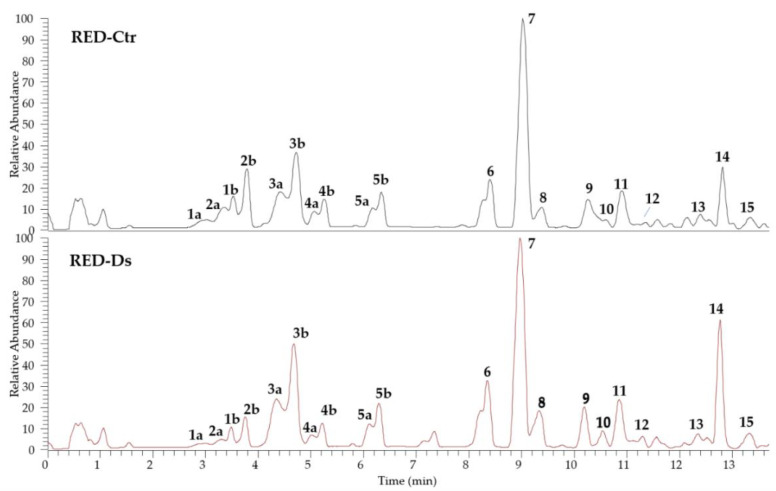
UHPLC-HR-ESI-MS profiles of tomato peel extracts of plants grown in normal (RED-Ctr) and in drought stress (RED-Ds) conditions.

The identification of the phenolic compounds in tomato peel from plants grown in normal (RED-Ctr) and in drought stress (RED-Ds) conditions was performed by UHPLC analyses, coupled to ESI-HR-MS. The chromatograms shown in Figure 2 evidenced a very similar profile with 15 identified phenol derivatives. Both extracts are rich in phenolic acids (compounds **1**–**5**, **11**, and **13**) and flavonoids (compounds **6**–**10**, **12**, **14**, and **15**). All compounds were identified by comparison of full MS and fragmentation patterns with literature data, except for chlorogenic acid (**3a** and **3b** isomers), rutin (**7**), and naringenin (**9**), confirmed by injection of reference standards (Table 1). Results were in agreement with the phenol composition in tomato peels previously reported [21,22,23].

Phenolic acids are caffeoyl/*p*-coumaroyl glucosides and caffeoylquinic derivatives, all revealed as *cis*/*trans* isomers. Among flavonoids, flavonol glycosides, having quercetin and kaempferol as aglycones, were found, as well as flavanone derivatives with naringenin as aglycone. 

Results from quantitative analyses (Table 2) showed a higher total phenol content in the control extract (1278 ± 28 µg/100 g FW) compared to that derived from plants grown in drought stress conditions (1073 ± 9 µg/100 g FW). Interestingly, even though the total phenols decreased in RED-Ds extract, the total content of phenolic acids is higher compared to the RED-Ctr. Indeed, the reduction seems to be due to the variation, especially in naringenin content (556 ± 2 vs. 793 ± 19 µg/100 g FW) and its derivatives.

### 2.2. HSMM Differentiation into Multinucleated Myotubes

**Figure 3:**
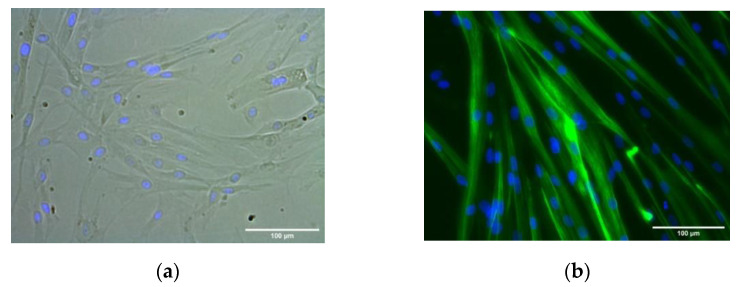
HSMM differentiation into multinucleated myotubes. Representative phase contrast (**a**) and dark-field images (**b**) of MYH2-immunostained cells (green), with Hoechst-labeled nuclei (blue) after 1 day growth medium (phase contrast image, (**a**)) and 3 days in the differentiation medium (dark-field image, (**b**)). (Images at ×20 magnification; *scale bar* = 100 microns).

Primary HSMM demonstrated robust myotube differentiation in accord with Owens et al. [24]. Cells were used at passage 5 for differentiation. Results showed that HSMM differentiated into MYH2-positive multinucleated myotubes (green) within 3 days in the differentiation medium (Figure 3). Myotubes were classified as elongated structures containing three or more nuclei within a single membrane structure. Irregular mass, clumps, or multi-branched aggregation conformations (complex dysmorphic myotubes) with three or more nuclei were not counted as myotubes. In HSMM myotubes, the nuclei tend to be arranged as singlets, or small groups, in linear arrays.

RNA was prepared from cells harvested on day 3 of culture in the differentiation medium, in order to further characterize the differentiation process of HSMM. Gene expression analyses were performed using quantitative RT-PCR for the late markers of muscle cell differentiation, such as MYH2, TNNT and MYOG [24,25]. The expression of the reference genes studied was detected in all samples. To dissect the best group of reference genes for the normalization of RT-PCR data, the stability was evaluated. The three most stable genes were: eEF1a, RPL13a and B2M (Avg M value = 0.269). 

**Figure 4:**
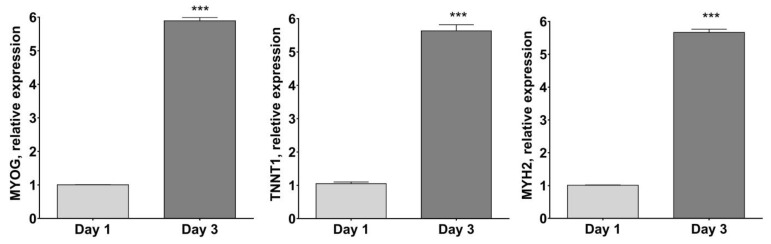
Skeletal muscle cell marker gene expression by RT-PCR. Plots show gene expression of late muscle cell markers Myosin heavy chain-2 (MYH2), Troponin T (TNNT1) and Myogenin (MYOG), normalized to three most stable reference genes, eEF1a, RPL13a and B2M, (Avg M value = 0.269) and then the 2^−ΔΔCt^ algorithm was applied for relative quantification. *** *p* < 0.001 vs. day 1 using Student’s *t*-test.

As reported in Figure 4, MYOG, TNNT1 andMYH2 mRNA expression resulted in significantly higher differentiated human myotubes after 3 days of culture in the differentiation medium (*p* < 0.001 vs. day 1). 

### 2.3. In Vitro Sarcopenia Induction

**Figure 5:**
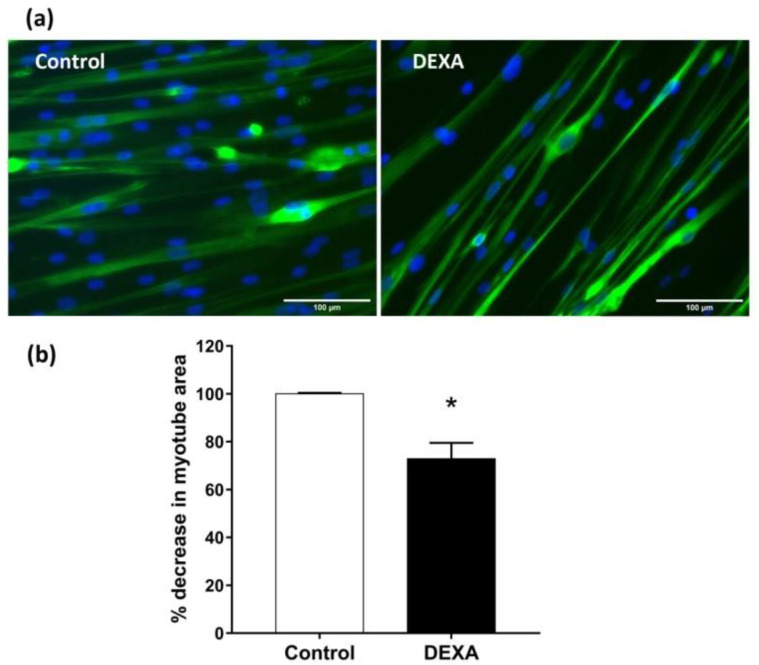
Induction of atrophy in HSMM myotubes. (**a**) Differentiated myotubes were treated with 50 µM of DEXA for 48 h. Representative images of MYH2/Hoechst-labeled myotube (green/blu). (**b**) Plots of the percent decrease, from untreated cells, in myotube area, are shown. Plots represent means ± SEM, *n* = 10. * *p* < 0.05 vs. untreated cells (control) using Student’s *t*-test. (Images at ×20 magnification; *scale bar* = 100 microns).

DEXA was used to induce myotubes atrophy [24], simulating sarcopenia. In particular, myotubes derived from HSMM were treated with DEXA (50 µM) for 48 h and then fixed and immuno-stained for MYH2. Nuclei were visualized by Hoechst staining. Total myotube areas were measured as described by Lecomte et al. [26] and results were plotted as the percent decrease in myotube area compared to untreated control cultures. Representative images of untreated myotubes or those treated with DEXA are shown in Figure 5a. HSMM myotubes undergo an atrophy-like response to DEXA treatment (Figure 5b, *p* < 0.05 vs. untreated cell). 

### 2.4. Tomato Peel Extracts Effect on Induced-Sarcopenia

Differentiated myotubes were treated for 2 h with tomato peel extracts, obtained from plants grown in normal (RED-Ctr) or in drought stress condition (RED-Ds), and then with DEXA for 48 h. Ascorbic acid (Asc) was used as the positive control [27]. The tomato peel extract concentration used in the present work was the same used as in our recent work [20], due to their non-cytotoxic activity and high antioxidant properties. 

**Figure 6:**
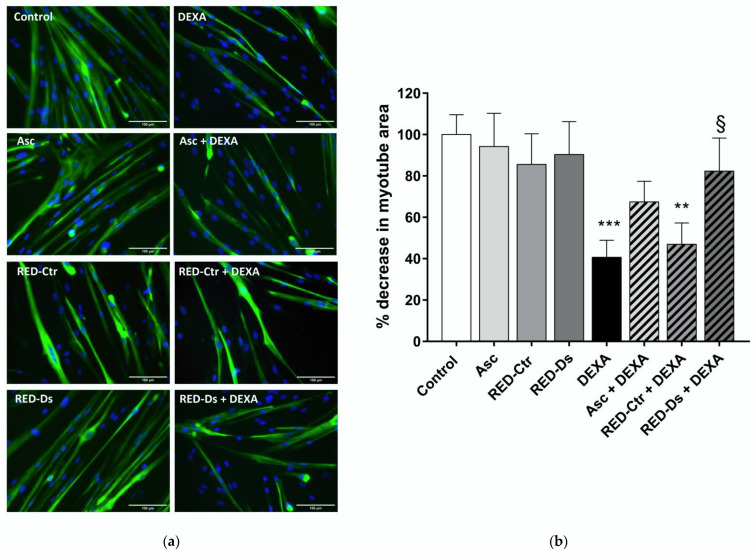
Tomato peel extract effect on myotube atrophy. (**a**) Representative images of differentiated myotubes (MYH2-positive cells (green), with Hoechst-labeled nuclei in blue) after 48 h of treatment with 50 µM DEXA or 5 µg/mL TPC tomato peel extracts (RED) of plants grown in normal (Ctr) or drought stress (Ds) conditions. Ascorbic acid (Asc) was used as positive control. All images at ×20 magnification; *scale bar* = 100 microns. (**b**) Plot shows the percent decrease, from untreated cultures, in myotube area at each treatment condition. Data represent means ± SEM, *n* = 10. ** *p* < 0.005 and *** *p* < 0.001 vs. untreated cell (control); § *p* < 0.05 vs. DEXA using one-way ANOVA.

Total myotube areas were measured as a percent decrease in untreated myotube area, in order to evaluate muscle atrophy. Results are reported in Figure 6. Data showed that both RED-Ds and RED-Ctr alone do not alter myotube area compared to untreated cells. Moreover, 5 µg GAE/mL TPC of RED-Ds extract showed a protective effect on muscle atrophy, induced by 50 µM DEXA (Figure 6b). As showed in the bar graph, DEXA induced statistically significant decreases in myotube area over untreated control cultures. When cells were pre-treated for 2 h with RED-Ds and then co-incubated with DEXA for 2 days, the decrease in myotube area induced by DEXA was not observed.

**Figure 7:**
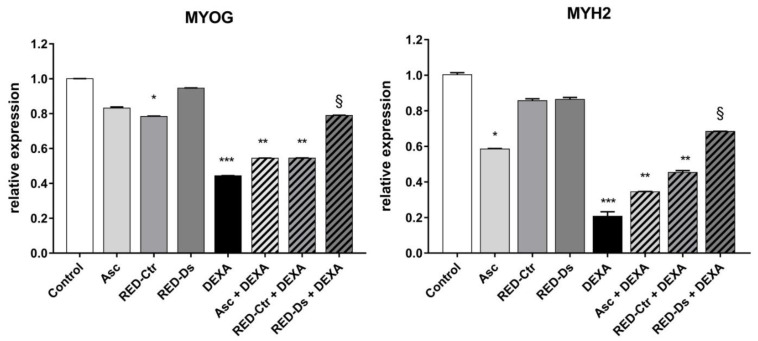
Gene expression of late Myosin heavy chain-2 (MYH2) and Myogenin (MYOG) muscle cell markers, normalized to three most stable reference genes, eEF1a, RPL13a and B2M, (Avg M value = 0.269). The 2^−ΔΔCt^ algorithm was applied for relative quantification in differentiated myotubes. Differentiated myotubes were treated with 50 µM of DEXA or with 5μg GAE/mL (TPC) of ascorbic acid (Asc) and Rosso di Pitigliano (RED) extracts of plants grown in normal (RED-Ctr) or in drought stress conditions (RED-Ds), for 48 h. Data show relative expression to the untreated cells (control). * *p* < 0.05, ** *p* < 0.005 and *** *p* < 0.001 vs. control; § *p* < 0.05 vs. DEXA using one-way ANOVA.

The expression level of late markers of differentiation myogenin and myosin heavy chain-2 were evaluated, in order to confirm the effects of tomato peel extracts on muscle atrophy. To this end, cell lysates were prepared at the end of the 48-h treatment period and mRNA expression for markers of muscle cell differentiation was measured by RT-PCR at 6 days of differentiation (Figure 7). 

The gene expression levels of late differentiation markers did not change when cells were treated with the extracts alone, except for RED-Ctr, which decreased MYOG expression, but not MYH2 expression, compared to untreated cells. These results indicate that polyphenols’ composition of tomato peel extract, grown in different conditions, can influence cell muscle differentiation. When the cells were also treated with DEXA, the RED-Ds extract showed a protective effect on cell differentiation (*p* < 0.05 vs. DEXA).

**Figure 8:**
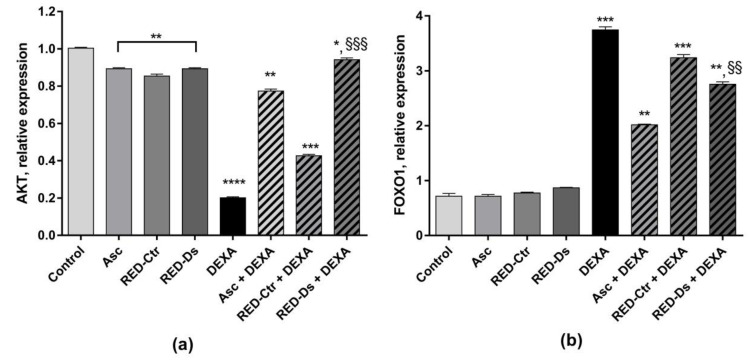
Protein kinase B (AKT) (**a**) and Forkhead BoxO1 (FOXO1) (**b**) mRNA expression in differentiated myotubes. Differentiated myotubes were treated with 50 µM of DEXA or with 5 μg GAE/mL (TPC) of ascorbic acid (Asc), Rosso di Pitigliano (RED) extracts of plants grown in normal (RED-Ctr) or in drought stress conditions (RED-Ds), for 48 h. Data show markers relative expression to the untreated cells (control). * *p* < 0.05, ** *p* < 0.005, *** *p* < 0.001 and **** *p* < 0.0001 vs. control; §§ *p* < 0.005 and §§§ *p* < 0.001 vs. DEXA using one-way ANOVA.

Akt and FOXO1 mRNA expression was also measured. As shown in Figure 8, Akt expression was significantly decreased by DEXA and RED-Ctr + DEXA treatments (*p* < 0.0001 and *p* < 0.001 vs. control, respectively). On the contrary, FOXO1 mRNA expression was significantly increased by DEXA treatment, as well as by RED-Ctr + DEXA treatment, compared to the untreated cells (*p* < 0.001). 

When cells were treated with RED-Ds or Asc, the negative effect of DEXA on Akt mRNA expression was reduced and a significant decrease in FOXO1 mRNA expression was observed (*p* < 0.005 vs. DEXA), (Figure 8a). 

## 3. Discussion

Tomato is one of the main sources of valuable nutrients in the Mediterranean diet, but is also a valuable nutrient all around the world. During tomato processing, obtained by-products are unused, but these by-products contain a great variety of biologically active substances with beneficial properties [28]. The tomato variety, Rosso Di Pitigliano, was chosen by the Tuscan Regional Bank of the Germplasm to evaluate the peel extract’s properties. In our recent study, the antioxidant properties of Rosso Di Pitigliano peel extract of plants grown in stress conditions on endothelial cells were demonstrated [20].

In the present study, for the first time, the effect of tomato by-products on a primary human skeletal muscle cells line, subjected to an atrophy stimulus, was evaluated. 

It is well known that DEXA induces muscle atrophy [8,9,12,29] and it can be used as a model of sarcopenia in vitro [30]. However, glucocorticoids are widely used to treat numerous inflammatory diseases; thus, a strategy to reduce collateral effects can be useful. Polyphenols have been reported to exert strong anti-atrophic effects by modulating the pro-atrophic factors or signaling pathways that contribute to muscle atrophy [15,31]. 

The authors observed that in tomato peel extracts, the differences between the total content of phenols detected with LC-MS are not excessively different. However, the variation within the classes of compounds is interesting. In particular, phenolic acids content increased in the stressed tomato peel extract, while some flavonoids, such as naringenin, decreased. 

Phenolic acids are important bioactive compounds of the polyphenolic class. They consist of an aromatic ring, with several hydroxyl groups attached. Some representative phenolic acids have been described to exert beneficial effects on muscles by promoting their growth or reducing their wasting by improving mitochondrial quality [31]. Our results showed that the tomato peel polyphenols of plants grown in drought stress conditions prevented DEXA-induced muscle atrophy, modulating expression of the genes involved in cell muscle metabolism. Chlorogenic acid, a phenolic acid compound obtained through the esterification of caffeic and quinic acids, is the main component of RED-Ds extract. This phenolic acid is abundant in some fruits, dietary vegetables, and daily beverages, such as coffee, pineapple, beans, strawberries, and apples [32]. Previous reports have demonstrated that chlorogenic acid exhibits a wide range of pharmacological effects, including anti-inflammatory, anti-oxidative, and anti-carcinogenic activities [33,34,35]. Moreover, as reported by Ommati et al., chlorogenic acid significantly improves skeletal muscle strength by promoting mitochondrial function [36]. The results of the present work suggest it provides a major contribution to the effects of the extracts that can be added to the effect of both flavonoids (rutin and quercetin) [15,37]. Recently, Chang et al. [37] reported that rutin up-regulates not only mitochondrial biogenesis and oxidative phosphorylation, but also myosin heavy chain content in C2C12 myotubes. Thus, despite the presence of *p*-coumaric acid glucoside, which is reported to inhibit horse-serum-induced skeletal muscle differentiation [38], RED-Ds does not reduce cell differentiation. Furthermore, RED-Ctr was shown to reduce MYOG expression and does not protect cells from DEXA treatment. This result could be due to the higher concentration of naringenin, the most abundant flavonoid, compared to that of the RED-Ds extract. As reported by Pellegrini et al. [39], naringenin delays skeletal muscle differentiation by blocking ERα-mediated Akt phosphorylation [39].

In the present study, the mRNA expression of Akt and FOXO1 was also evaluated. Glucocorticoids exert their physiological actions mainly via a nuclear pathway to directly affect target gene transcription. In particular, DEXA exerts its biological effects predominantly via the glucocorticoid receptor. DEXA stimulation could be mediated by the increased expression of other transcription factors and associated coactivators that, in turn, bind to and activate the MuRF1 promoter. Recently, the FOXO family (FOXO1, FOXO3a, and FOXO4) have been implicated as key regulators of gene expression during skeletal muscle atrophy [11,40]. FOXO1 mRNA, in particular, is up-regulated during DEXA treatment [41,42]. In our study, we observed that RED-Ds, but not RED-Ctr, can modulate FOXO1 mRNA expression, down-regulating their expression in the presence of DEXA. This result could be due to the effect of naringenin, the most abundant flavonoid found in RED-Ctr and involved in the delays in skeletal muscle differentiation. However, further studies are needed to understand the specific role of tomato polyphenols in muscle atrophy.

As recently reported by Conti et al. [43], water shortage stress can affect the yield of tomato crops, the volume, diameter and composition of the fruits, as well as the plant metabolic pathways, increasing bioactive production. According to Sanchez et al. [44], when the plant is stressed, it activates the simpler metabolic pathways, which leads to simple phenolic compounds, rather than the mixed pathway that leads to flavonoids. 

## 4. Materials and Methods

### 4.1. Cells, Media and Supplement

Human skeletal muscle myoblasts (HSMM, Catalog #CC-2580, lot #0000655307, Lonza, Walkersville, MD, USA), isolated from the quadriceps muscle of an 18-year-old male and Skeletal Muscle Growth Media-2 (SKGM-2 Medium) with single quots kit, were purchased from Lonza (Walkersville, MD, USA). HSMM differentiation medium was prepared by adding 2% horse serum to DMEM-F12 medium (both from Lonza, Walkersville, MD, USA). 

Anti-skeletal myosin Fast primary antibody (MY-32); Dexamethasone (DEXA); bovine serum albumin (BSA); formalin, Triton X-100 and Hoechst nuclear stain (#33342) were purchased from Sigma-Aldrich (Milan, Italy). Alexa 488 conjugated anti-mouse IgG secondary antibody was purchased from Invitrogen (Thermo Fischer Scientific, Monza, Italy). Methanol, formic acid, and water for UHPLC analyses were purchased from Romil-Deltek (Pozzuoli, Italy). Chlorogenic acid (purity ≥ 95%), rutin (purity ≥ 95%), and naringenin (purity ≥ 95%), used as reference standards were purchased from Sigma-Aldrich (Milan, Italy).

### 4.2. Sample Preparation

#### 4.2.1. Fruit Harvesting and Growth Conditions

The tomato variety used for the study was chosen from nine local Tuscan varieties (*Solanum lycopersicum* L.) registered at the Tuscan Regional Germplasm Bank, characterized by different morphological and agronomic features. The tomato variety used for this study was Rosso di Pitigliano obtained by growing plants in normal conditions or in drought stress conditions. Plants were harvested and stressed as described by Cesare et al. [20].

#### 4.2.2. Tomato Peel Extracts Preparation

Lyophilized tomato peel sample was prepared as previously described by Cesare et al. [20]. The freeze-dried tomato peel extracts were transferred into airtight containers and stored at −20 °C until used. 

### 4.3. UHPLC-HR-ESI-MS Analyses of Tomato Peel Extracts

Tomato peel extracts were analyzed by means of UHPLC using a Vanquish Flex Binary pump LC system coupled with an ESI-HR-MS, Q Exactive Plus MS, Orbitrap-based FT-MS system (Thermo Fischer Scientific Inc., Bremen, Germany). Each sample was injected (5 µL) on a C-18 Kinetex^®^ Biphenyl (Phenomenex, Bologna, Italy) column (100 *×* 2.1 mm, 2.6 μm particle size) provided by a Security Guard TM Ultra Cartridge (Phenomenex, Bologna, Italy). A linear solvent gradient was developed for elution using formic acid in MeOH 0.1% *v*/*v* (solvent A) and formic acid in H_2_O 0.1% *v*/*v* (solvent B), from 5 to 55% A within 14 min, at a flow rate 0.5 mL/min. The autosampler and column oven temperatures were maintained at 4 and 35 °C, respectively. HR mass spectra were acquired in a scan range of *m/z* 250–1200 in ESI negative ion mode, by using ionization parameters as previously reported [45] operating in full (70000 resolution, 220 ms maximum injection time) and data-dependent-MS/MS scan (17500 resolution, 60 ms maximum injection time). 

The quantitative analysis of phenols was performed by constructing calibration curves based on rutin (concentration range 0.1–50 μg/mL), naringenin (concentration range 0.1–100 μg/mL), and chlorogenic acid (concentration range 5–500 μg/mL), as external standards for flavanol glycosides, flavanones, and phenolic acids, respectively. Standard methanol solutions were prepared in triplicate at different concentrations by serial dilution starting from a stock solution of 1.0 mg/mL. The calibration curve was obtained by using concentration (µg/mL) with respect to the areas obtained by integration of full MS peaks of the reference standards. Linear simple correlation was used to analyze the relation between variables (*R*^2^ = 1 for rutin and naringenin; *R*^2^ = 0.9989 for chlorogenic acid). The phenol amount was obtained by using a Microsoft^®^ Office Excel (Redmond, Washington, USA) and expressed as µg of 100 g of FW. 

### 4.4. Cell Culture and Differentiation

HSMM was expanded in complete growth medium and incubated in a humidified incubator at 37 °C, 5% CO_2_. Culture medium was changed every three days. Cells were passaged when they reached 50–70% confluency. 

To induce differentiation, cells were plated at 20,000 cells/cm^2^ in 12-well polystyrene cell culture plates and incubated overnight in growth medium in a cell culture incubator (37 °C, 5% CO_2_). The following morning, the growth medium was replaced with differentiation medium and the cultures were incubated for 3 days, during which time myotube differentiation occurred.

### 4.5. Cell Treatment in Sarcopenia-Induced Model

The cellular model of sarcopenia was reproduced by treating HSMM with 50 µM DEXA on day 3 of differentiation. On the same day, 5 µg GAE/mL TPC of RED-Ctr, 5 µg GAE/mL TPC of RED-DS and 5 µg Asc were added 2 h prior to DEXA treatment and cells were incubated for another 2 days. Cells were then treated for immunostaining measurement. For gene expression studies, the cell pellet was prepared and stored at −80 °C in a local refrigerator.

### 4.6. Immunostaining and Fluorescence Analysis

The HSMM differentiation into multinucleated myotubes was assessed by immunofluorescent techniques. Briefly, after treatment cells were fixed with 10% formalin for 20 min at room temperature (RT). Cells were washed three times with PBS, and then permeabilized with 0.5% Triton X-100 at RT for 15 min. Non-specific binding sites were blocked by incubation with 2% BSA, 0.25% Triton X-100 in PBS at RT for 30 min.

Cells were then incubated with anti-skeletal myosin FAST primary antibody (MY-32) diluted 1:500 in blocking buffer at RT for 2 h, to evaluate MYH2-positive cells. After rinsing with blocking buffer without Triton X-100 for 10 min, cells were incubated with Alexa 488 (green) conjugated anti-Mouse IgG secondary antibodies at 1:400 dilution along with Hoechst nuclear stain at 1:5,000 dilution in blocking buffer at RT for 1 h. 

Cells were washed with PBS three times, 5 min each, and viewed under a fluorescence microscope (Nikon Eclipse Ti, Amsterdam, The Netherlands) equipped with a digital CCD camera and 20X objective. Images were recorded using AxioVision (Carl Zeiss MicroImaging, GmbH) software.

Total myotube areas were measured as described by Semsarian et al. [46] and Lecomte et al. [26] and results were plotted as the percent decrease or increase in myotube area compared to untreated control cultures.

### 4.7. RNA Extraction, Reverse Transcription and Real-Time PCR Experiments

Total RNA was extracted from cultured cells with High Pure RNA Isolation Kit (Roche Diagnostics Indianapolis, USA). RNA concentration was determined by Nanodrop 1000 (Thermo Fisher Scientific) at 260 nm. The ratio of readings at 260 nm and 280 nm (A260/A280) provided an estimate for the purity of RNA and only samples that showed OD 260/280 ratios of 1.9–2.1 were used. The RNA samples were stored at −80 °C for use in gene expression studies. First strand cDNA was synthesized with iScript cDNA Synthesis kit (Bio-rad, Hercules, CA, USA), following the manufacturer’s protocol. The cDNA samples obtained were placed on ice and stored at 4 °C until further use. 

Primer pairs of both interested markers and reference genes were designed with Primer-BLAST (https://blast.ncbi.nlm.nih.gov/Blast.cgi?PROGRAM=blastn&PAGE_TYPE=BlastSearch&LINK_LOC=blasthome, accessed on 23 May 2020) and synthesized by Merck Life Science S.r.l. Reaction conditions for all primers were optimized by means of a gradient PCR, conducted to assess the optimal annealing temperature, while a standard curve obtained by scalar dilution of a cDNA pool was always generated to verify PCR efficiency. In Table 3 the primer sequence details of the analyzed genes are reported. RT-PCR reactions were performed in duplicate in the Bio-Rad CFX Connect Thermal Cycler (CFX-Connect Real-Time PCR detection systems, Bio-Rad). EvaGreen (SsoFAST EvaGreen Supermix, Bio-Rad) was used for monitoring cDNA amplification. PCR was performed in a volume of 20 μL per reaction, including 0.2 μM of each primer (Merck Life Science S.r.l., Milan, Italy), samples, reagent, and sterile H_2_O. RT-PCR data were normalized with three most stable reference genes, eEF1a, RPL13a and B2M, (Avg M value = 0.269) and then the 2^−ΔΔCt^ algorithm was applied for relative quantification using Bio-Rad’s CFX Maestro software (CFX Real-Time PCR detection systems, Bio-Rad Laboratories Inc., Hercules, CA, USA).

### 4.8. Statistical Analysis

Results are expressed as means and standard error of mean (SEM). Statistical analysis was carried out using Student’s *t*-tests or one-way ANOVA using GraphPad PRISM 8 (GraphPad software, Inc., La Jolla, CA, USA) software for Windows. All tests were considered significant when *p* < 0.05.

## 5. Conclusions

The present paper showed, for the first time, to the best of the authors’ knowledge, that tomato by-product extracts, obtained from plants grown in drought stress conditions, reduced DEXA-induced atrophy in human skeletal muscle cells. These results encourage further studies for a potential use of tomato peel extracts in the development of anti-atrophic food supplements used for neutralizing pharmacological or oxidative-stress-induced pathological conditions in skeletal muscles. Nevertheless, the protective effect of the peel extracts could depend on their qualitative polyphenolic composition. The chemical characterization of the polyphenolic composition that is responsible for a strong anti-atrophic activity would help to assess the quality of extracts intended for use as supplements.

## Figures and Tables

**Table 1:** UHPLC-HR-ESI-MS/MS data of phenols detected in peel extracts of plants grown in normal (RED-Ctr) and in drought stress (RED-Ds) conditions.

Peak ^a^	Compound ^b^	*t*_R_ (min)	HR-[M−H]^−^ (*m/z*)	HR-MS/MS Product Ions (*m/z*) ^c^	Molecular Formula	Error (ppm)	RED Extract
	Phenolic acids						
**1a**	Caffeic acid glucoside isomer I	3.0	341.0875	**179.03**, 145.03, 161.02, 135.04	C_15_H_18_O_9_	−0.73	Ctr, Ds
**2a**	*p*-Coumaric acid glucoside isomer I	3.3	325.0927	**163.04**, 119.05, 93.03	C_15_H_18_O_8_	−0.31	Ctr, Ds
**1b**	Caffeic acid glucoside isomer II	3.5	341.0875	**179.03**, 145.03, 161.02, 135.04	C_15_H_18_O_9_	−0.73	Ctr, Ds
**2b**	*p*-Coumaric acid glucoside isomer II	3.8	325.0927	**163.04**, 145.03, 119.05, 93.03	C_15_H_18_O_8_	−0.31	Ctr, Ds
**3a**	Chlorogenic acid isomer I ^d^ (3-*O*-caffeoylquinic acid)	4.4	353.0878	**191.06**, 179.03, 173.04, 135.04	C_16_H_18_O_9_	0.00	Ctr, Ds
**3b**	Chlorogenic acid isomer II ^d^	4.7	353.0878	**191.06**, 179.03, 173.04, 135.04	C_16_H_18_O_9_	0.00	Ctr, Ds
**4a**	*p*-Coumaric acid glucoside isomer III	5.1	325.0927	**163.04**, 145.03, 119.05, 93.03	C_15_H_18_O_9_	−0.31	Ctr, Ds
**4b**	*p*-Coumaric acid glucoside isomer IV	5.2	325.0927	**163.04**, 145.03, 119.05, 93.03	C_15_H_18_O_9_	−0.31	Ctr, Ds
**5a**	Caffeoylquinic acid isomer I	6.2	353.0878	**191.06**, 179.03, 135.04	C_16_H_18_O_9_	0.00	Ctr, Ds
**5b**	Caffeoylquinic acid isomer II	6.3	353.0878	**191.06**, 179.03, 135.04	C_16_H_18_O_9_	0.00	Ctr, Ds
**11**	Dicaffeoylquinic acid isomer I	10.8	515.1191	**353.09**, 191.06, 179.03, 173.04, 135.04	C_25_H_24_O_12_	−0.78	Ctr, Ds
**13**	Dicaffeoylquinic acid isomer II	12.4	515.1191	**353.09**, 191.06, 179.03, 173.04, 135.04	C_25_H_24_O_12_	−0.78	Ctr, Ds
	Flavonoids						
**6**	Quercetin 3-*O*-(2″-*O*-apiofuranosyl-6″-*O*-rhamno pyranosyl- glucopyranoside)	7.3	741.1885	**300.03**, 301.02, 271.03	C_32_H_40_O_21_	+0.13	Ctr, Ds
**7**	Rutin ^d^	9.0	609.1461	**300.03**, 301.02, 271.03	C_27_H_30_O_16_	0.00	Ctr, Ds
**8**	Kaempferol rutinoside-pentoside	9.3	725.1932	284.03, **285.04**, 255.03	C_32_H_38_O_19_	−0.28	Ctr, Ds
**9**	Kaempferol 3-*O*-rutinoside	10.2	593.1509	**284.03**, 285.04, 255.03	C_27_H_30_O_15_	−0.34	Ctr, Ds
**10**	Naringenin 7-*O*-glucoside	10.5	433.1138	**271.06**, 151.00, 119.05	C_21_H_22_O_10_	−0.46	Ctr, Ds
**12**	Naringenin chalcone glucoside	11.3	433.1138	**271.06**, 151.00, 119.05	C_21_H_22_O_10_	−0.46	Ctr, Ds
**14**	Naringenin ^d^	12.8	271.0611	151.00, 119.05	C_15_H_12_O_5_	−0.37	Ctr, Ds
**15**	Naringenin calchone	13.4	271.0611	151.00, 119.05	C_15_H_12_O_5_	−0.37	Ctr, Ds

^a^ Compound numbers correspond with peak numbers in Figure 2. ^b^ Tentatively identified based on MS/MS and literature data. ^c^ The ion base peaks are shown in bold. ^d^ Confirmed by reference standard.

**Table 2:** Amount of constituents detected in peel extracts of plants grown in normal (RED-Ctr) and in drought stress (RED-Ds) conditions.

Peak ^a^	Compound	RED-Ctr (µg/100 g FW ± SD)	RED-Ds (µg/100 g FW ± SD)
	Phenolic acids		
**1a + 1b**	Caffeic acid glucoside (isomers I and II)	8.18 ± 0.1	18.6 ± 0.1 *
**2a + 2b**	*p*-Coumaric acid glucoside (isomers I and II)	12.8 ± 0.1	37.8 ± 0.1 *
**3a + 3b**	Chlorogenic acid (isomers I and II)	46.9 ± 0.5	51.9 ± 0.9 *
**4a + 4b**	*p*-Coumaric acid glucoside (isomers III and IV)	9.13 ± 0.1	16.3 ± 0.2 *
**5a + 5b**	Caffeoylquinic acid (isomers I and II)	15.0 ± 0.2	18.3 ± 0.2 *
**11**	Dicaffeoylquinic acid (isomer I)	16.5 ± 0.4	20.5 ± 0.4 *
**13**	Dicaffeoylquinic acid (isomer II)	3.47 ± 0.06	5.03 ± 0.1 *
	Flavonoids		
**6**	Quercetin 3-*O*-(2″-*O*-apiofuranosyl-6″-*O*-rhamnopyranosyl-glucopyranoside)	16.1 ± 0.4	16.7 ± 0.4
**7**	Rutin	45.5 ± 0.5	62.3 ± 0.9 *
**8**	Kaempferol rutinoside-pentoside	4.88 ± 0.3	5.04 ± 0.2
**9**	Kaempferol 3-*O*-rutinoside	7.23 ± 0.3	8.17 ± 0.2 *
**10**	Naringenin 7-*O*-glucoside	103 ± 4 *	90.0 ± 2
**12**	Naringenin chalcone glucoside	119 ± 1 *	109 ± 1
**14**	Naringenin	793 ± 19 *	556 ± 2
**15**	Naringenin chalcone	77.4 ± 2 *	58.0 ± 0.6
	Total phenolic acids	112 ± 1	168 ± 2 *
	Total flavonoids	1166 ± 27 *	905 ± 7
	Total phenols	1278 ± 28 *	1073 ± 9

^a^ Compound numbers correspond to the peak numbers in Figure 2 and Table 1. * Statistically significant differences between the samples determined by the *t*-test, *p* < 0.01.

**Table 3:** Beta-2 Microglobulin (B2M); Eukaryotic translation elongation factor 1 alpha (eEF1A); Forkhead Box O1 (FOXO1); Myogenin (MYOG); Myosin heavy chain 2 (MYH2); Protein kinase B (AKT); Ribosomal protein L13a (RPL13A); Troponin T (TNNT1).

Gene	Sequence		GenBank, Accession	Length (bp)	Temperature (°C)
**AKT**	Forward	CTGCACAAACGAGGGGAGTA	NM_001014431.2	142	60
	Reverse	GCGCCACAGAGAAGTTGTTG			
**B2M**	Forward	CACTGAATTCACCCCCACTGA	NM_004048.4	102	60
	Reverse	GCTTACATGTCTCGATCCCAC			
**eEF1A**	Forward	CTTTGGGTCGCTTTGCTGTT	NM_001402	183	60
	Reverse	CCGTTCTTCCACCACTGATT			
**FOXO1**	Forward	GGGTTAGTGAGCAGGTTACAC	NM_002015.4	170	60
	Reverse	CTTTGCTGCCAAGTCTGACG			
**MYH2**	Forward	CTCAAAGCTCTCTGCTACCCC	NM_017534.6	88	60
	Reverse	CTACTGCGTTGGACACCTGTTCT			
**MYOG**	Forward	AGATTGTCTTCCAAGCCGGG	NM_002479.6	112	60
	Reverse	CTGGCTTCCTAGCATCAGGG			
**RPL13A**	Forward	CGCCCTACGACAAGAAAAAG	NM_012423	206	60
	Reverse	CCGTAGCCTCATGAGCTGTT			
**TNNT1**	Forward	GTCAGAGAGAGCCGAGCAAC	NM_001126133.3	197	60
	Reverse	CACGCTTCTGTTCTGCCTTG			

## Data Availability

Not applicable.

## Sample Availability

Samples of the compounds RED-Ds and RED-Ctr are available from the authors.
